# Supplementary material for: PGC-1α overexpression partially rescues impaired oxidative and contractile pathophysiology following volumetric muscle loss injury
Source: Sci Rep. 2019 Mar 11;9:4079. doi: 10.1038/s41598-019-40606-6 (PMC6411870; doi:10.1038/s41598-019-40606-6)
Supplement: Supplementary file 1 — Supplementary Information [file 41598_2019_40606_MOESM1_ESM.docx]

**PGC-1α overexpression partially rescues impaired oxidative and contractile pathophysiology following volumetric muscle loss injury**

William M. Southern^1,2^, Anna S. Nichenko^1,2^, Kayvan F. Tehrani^2^, Melissa J. McGranahan^1^, Laxminarayanan Krishnan^3^, Anita E. Qualls^1,2^, Nathan T. Jenkins^1^, Luke J. Mortensen^2^, Hang Yin^4,5^, Amelia Yin^4,5^, Robert E. Guldberg^6^, Sarah M. Greising^7^, Jarrod A. Call^1,2,*^

**Supplementary Information**


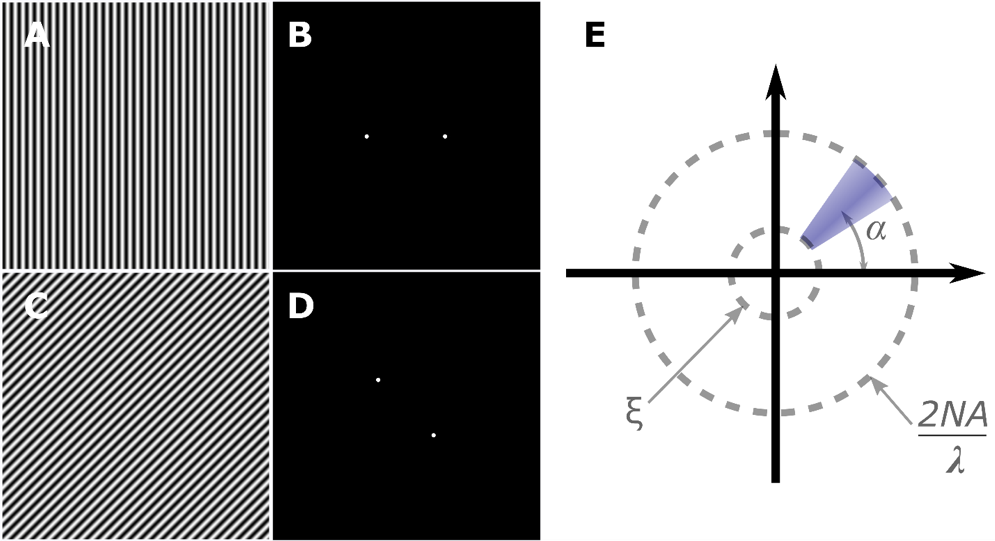


**Supplementary Figure 1:** Illustration of the angular Fourier filter. **A** and **C** show sinusoidal patterns in the spatial domain with frequency of ω at 0° and 45°, respectively. Corresponding power spectral density (PSD) of **A** and **C** are shown in **B** and **D**. In the PSD there exists only two peaks at the frequencies ω and -ω, with zero frequency being at the center. Because the angle of the pattern in the spatial domain image (**A**, **C**) are followed in the PSD (**B**, **D**) we can use this approach to characterize the angular distribution of patterned structures such as the mitochondrial network. Our Angular Fourier Filter is shown in **E**, depicting the gaussian wedge filter placed at angle α. It also acts as a bandpass filter to suppress features smaller than the diffraction limit to avoid unwanted noise, and larger than the mitochondrial network to avoid distortion of the analysis.
